# The *Listeria monocytogenes* Key Virulence Determinants *hly* and *prfA* are involved in Biofilm Formation and Aggregation but not Colonization of Fresh Produce

**DOI:** 10.3390/pathogens7010018

**Published:** 2018-02-01

**Authors:** Robert Price, Victor Jayeola, Jeffrey Niedermeyer, Cameron Parsons, Sophia Kathariou

**Affiliations:** Department of Food, Bioprocessing and Nutrition Sciences, North Carolina State University, Raleigh, NC 27604, USA; reprice2@ncsu.edu (R.P.); vjayeol@ncsu.edu (V.J.); janieder@ncsu.edu (J.N.); skathar@ncsu.edu (S.K.)

**Keywords:** *Listeria monocytogenes*, biofilm, aggregation, produce

## Abstract

*Listeria monocytogenes* has been extensively studied as a model facultative intracellular pathogen. While the roles of major virulence factors in host-pathogen interactions have been extensively characterized, recent work suggests that some of these factors can also contribute to environmental proliferation of this pathogen. In this study, we characterized two non-hemolytic transposon mutants of strain 2011L-2858 (serotype 1/2b), implicated in the 2011 listeriosis outbreak via whole cantaloupe, for their capacity to form biofilms on polystyrene, aggregate, and colonize cantaloupe rind. One mutant harbored a single *mariner*-based transposon insertion in *hly*, encoding the hemolysin Listeriolysin O, while the other harbored a single insertion in *prfA*, encoding PrfA, a master regulator for *hly* and numerous other virulence genes. Biofilm formation was significantly reduced in the *prfA* mutant, and to a lesser extent, in the *hly* mutant. Inactivation of either *hly* or *prfA* significantly reduced *L. monocytogenes* aggregation. However, both mutants adhered similarly to the wildtype parental strain on cantaloupe rind at either 25 or 37°C. Furthermore, growth and competitive fitness of the mutants on cantaloupe rind was not significantly impacted at either temperature. The findings suggest that, in spite of their involvement in biofilm formation and aggregation, these key virulence determinants may not be required for the ability of *L. monocytogenes* to colonize fresh produce.

## 1. Introduction

*Listeria monocytogenes* is a facultative intracellular pathogen responsible for human foodborne disease (listeriosis) associated with severe symptoms (e.g., septicemia, meningitis, stillbirths) and high case fatality rate [1,2]. Strains implicated in human listeriosis are primarily of three serotypes, 1/2a, 1/2b and 4b [3,4]. Even though foods such as soft cheeses, deli meats and processed seafood have been implicated in numerous outbreaks, in recent years produce has been increasingly recognized as a vehicle for human listeriosis [5,6]. In 2011, a large, multistate listeriosis outbreak in the United States was attributed to whole cantaloupe contaminated with strains of serotype 1/2a and 1/2b. The outbreak resulted in 147 reported cases of listeriosis and multiple deaths. This was the first listeriosis outbreak involving whole cantaloupe and the largest reported to date [7].

The *L. monocytogenes* hemolysin Listeriolysin O (LLO), encoded by *hly*, and PrfA, a master transcriptional activator for *hly* and numerous other virulence genes, are key virulence determinants of *L. monocytogenes*, extensively characterized for their roles in host-pathogen interactions [8,9,10,11]. However, PrfA has also been found to be required for biofilm formation by the serotype 1/2a strains 10403S and EGD [12,13], and mutants with deregulated, constitutive *prfA* expression (“PrfA*”) exhibited decreased competitive fitness upon exposure to several environmental stresses [14]. In addition, ActA, a virulence determinant under PrfA control and key to intracellular motility and cell-to-cell spread of *L. monocytogenes*, was shown to be required for *L. monocytogenes* EGD cell aggregation and biofilm formation [13]. Even though underlying mechanisms remain to be fully elucidated, such findings suggest that these virulence determinants also contribute to environmental adaptation of *L. monocytogenes* outside of the animal host. However, these studies were largely performed with reference strains such as 10403S and EGD which belong to clonal groups that seem to be under-represented in human disease [15] and may not fully reflect adaptive attributes of strains frequently implicated in listeriosis outbreaks, considering the increasingly important role of fresh produce in listeriosis outbreaks [5,6], similar studies with strains from produce-related outbreaks have been noticeably lacking. Potential links between *prfA* or key virulence determinants under PrfA control and the capacity of *L. monocytogenes* to colonize fresh produce also remain to be identified and investigated. The objective of the current study was to characterize the impact of *prfA* and *hly* on the ability of *L. monocytogenes* strain 2011L-2858, a serotype 1/2b strain associated with a major listeriosis outbreak involving contaminated whole cantaloupe in 2011, to form biofilms, aggregate, and colonize the surface of fresh cantaloupe.

## 2. Results and Discussion

The serotype 1/2b strain 2011L-2858, hereafter referred to as “2858”, belongs to clonal complex 5, and was implicated in the 2011 multistate outbreak of listeriosis [5,16]. Isolates of the same serotype and genotype contributed to 27% of the cases and 30% of the fatalities in this outbreak and were also recovered from whole fruit and the packing environment [5,7]. Screening of approx. 1900 transposon mutants of this strain on blood agar plates revealed three that were non-hemolytic. Among them, mutants B2G6 (insertion at nt 225 of *hly*) and J2E3 (insertion at nt 65 of *prfA*) were chosen for further work. Southern blots confirmed that each mutant harbored a single insertion of the transposon (data not shown). The mutants were indistinguishable from the parental strain and each other in motility (data not shown), colony or cell morphology, and growth rate in BHI at 28 or 37 °C (Supplemental Figures S1–S3).

### 2.1. hly and prfA are Required for Biofilm Formation of L. monocytogenes

**Figure 1:**
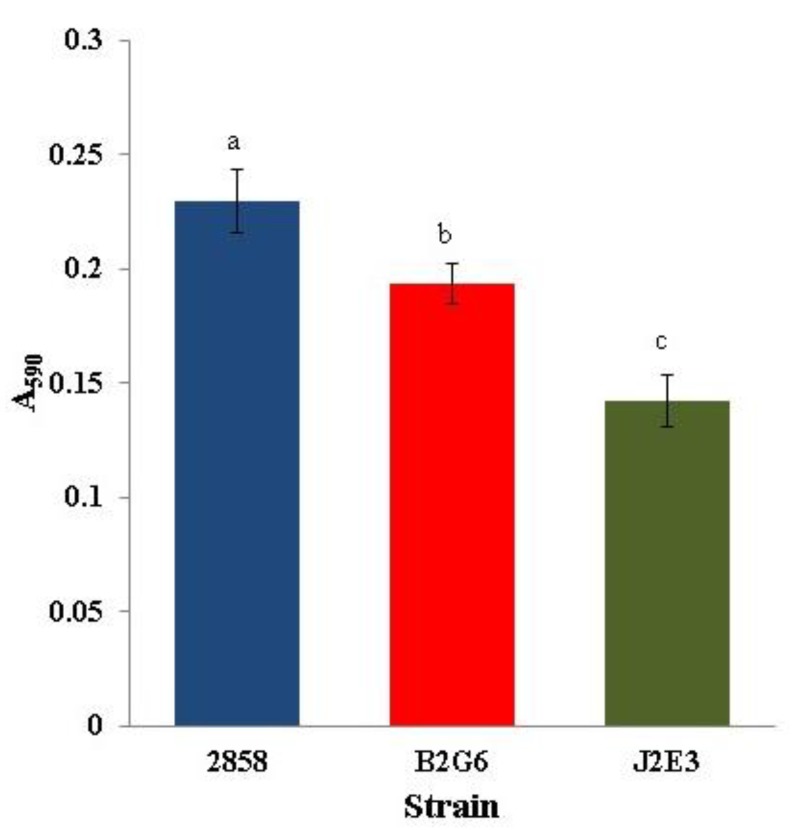
Impact of *hly* and *prfA* on biofilm formation. Biofilms were established at 37 °C in wells of 96-well polystyrene plates over 48 h (8 wells/strain) and measured following staining with crystal violet as described in Materials and Methods. Strains from this study are shown on the x axis, wild type 2011L-2858 (2858), *hly* inactivated mutant B2G6, and *prfA* inactivated mutant J2E3. The y axis indicates absorbance of ethanol used to destain crystal violet stained wells at 590 nm, thus serving as an indication of biofilm formation. Different letters indicate statistically significant differences (*p* < 0.05). Data are from three independent trials.

In spite of increasing interest in the interface between environmental biology and pathogenesis of *L. monocytogenes* [9,10,17,18,19] the potential roles of key virulence genes of *L. monocytogenes* in adaptations other than those directly associated with pathogenesis remain poorly understood. Previous studies with *L. monocytogenes* reference strains 10403S and EGD (both of serotype 1/2a) revealed a significant requirement of *prfA* for biofilm formation in polyvinyl 96-well plates (Lemon, Freitag and Kolter 2010; Travier et al., 2013). However, *hly* mutants of strain 10403S did not exhibit defects in biofilm formation [12], while *hly* inactivation of strain EGD resulted in moderate biofilm reductions [13]. In the case of strain 2858, both the *prfA* and *hly* deficient mutants exhibited significant (*p* < 0.05) reduction in biofilm (Figure 1). Inactivation of *prfA* was accompanied by a pronounced reduction in biofilm while the *hly* mutant exhibited moderate reduction, similarly to what was observed with strain EGD [13]. Such data suggest that, besides *hly*, additional determinants under PrfA transcriptional control are implicated in biofilm formation.

The master virulence regulator PrfA controls expression of several virulence genes, including *hly* and *actA*, with the latter having been shown to contribute significantly to *L. monocytogenes* aggregation and biofilm formation [13]. Such involvement of ActA on biofilm formation, together with the moderate impacts of *hly* inactivation observed here and by others [13], may contribute to the much more pronounced biofilm reductions exhibited by the *prfA* mutant J2E3 than by the *hly* mutant B2G6 (Figure 1).

### 2.2. Inactivation of Either prfA or hly Results in Decreased Aggregation

**Figure 2:**
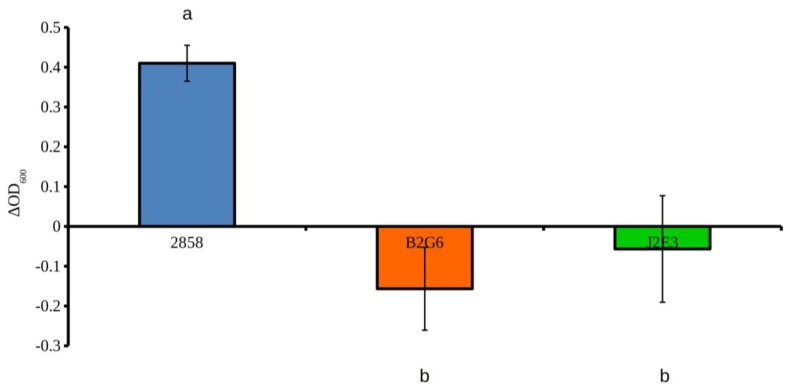
Impact of inactivation of either *hly* or *prfA* on cell aggregation. Aggregation assays of 2858 (wild type parental strain), *hly* mutant B2G6 and *prfA* mutant J2E3 were performed in BHI as described in Materials and Methods. Different letters indicate statistically significant differences (*p* < 0.05).

**Figure 3:**
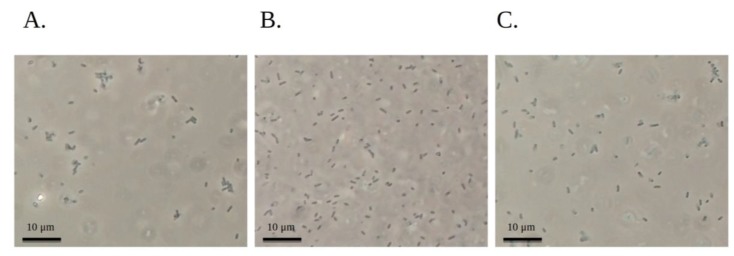
*L. monocytogenes* aggregation. Aggregation assays were performed in BHI at 37°C, and bacteria from approx. 1 cm below the surface were visualized with phase contrast microscopy as described in Materials and Methods. (**A**) Parental strain 2858; (**B**) *hly* mutant B2G6; (**C**) *prfA* mutant J2E3.

Aggregation in *L. monocytogenes* in vitro has been shown to require intact *prfA*; in a murine model it is associated with aggregation and persistence in the intestinal lumen and impacts the levels and length of *L. monocytogenes* shedding in feces [13]. Aggregation assays in our study showed that *prfA* inactivation significantly decreased cell aggregation in vitro at 37 °C (Figure 2 and Figure 3), in agreement with previous reports with strain EGD [13]. The differences in OD_600_ (Figure 2), which suggested differences in aggregative ability between wild type 2858 and both mutant strains were corroborated by phase contrast microscopy (Figure 3) which also revealed more wild type formed aggregates than either of the mutant strains. In strain EGD, the PrfA-controlled determinant ActA was shown to mediate aggregation via direct ActA-ActA interactions in the C-terminal region of the protein which is not responsible for actin polymerization and intracellular motility during infection [13]. Similar ActA-based interactions likely contribute to the role of *prfA* in aggregation of strain 2858, though analysis of *actA* mutants will be required for confirmation.

Surprisingly, inactivation of *hly* also had a pronounced impact on aggregation in strain 2858 (Figure 2 and Figure 3). This contrasted with strain EGD, where *hly* had only marginal involvement in aggregation [13]. The more noticeable involvement of *hly* in aggregation of strain 2858 in the current study may reflect strain-specific differences which remain to be characterized. In addition to having different serotypes, strains EGD and 2858 belong to different lineages (lineage II and I, respectively), and are thus likely to differ in multiple attributes. Molecular mechanisms mediating the role of LLO in aggregation in strain 2858 remain to be identified. It also remains to be determined whether *hly* has generally a more pronounced impact on aggregation in serotype 1/2b than serotype 1/2a, or in lineage I vs. lineage II. Lastly, the aggregation impact of the *prfA* mutation on other determinants besides *hly* that are under PrfA control remains to be determined in this strain.

### 2.3. Insertional Inactivation of hly or prfA does not Impact Growth and Adherence of L. monocytogenes on Cantaloupe Rind at 25 or 37 °C

**Figure 4:**
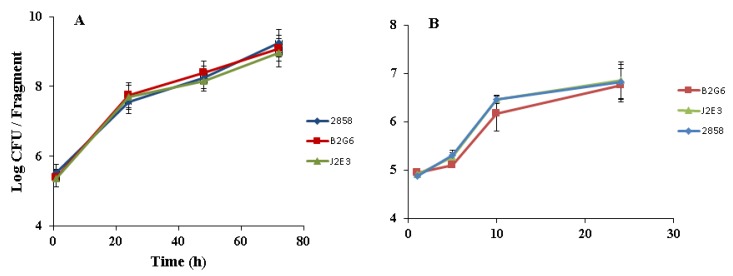
Growth of *hly* and *prfA* mutants on cantaloupe rind. Rind fragments were inoculated with approx. 10^5^ CFU of each strain and growth was determined at (**A**) 25 °C and (**B**) 37 °C as described in Materials and Methods. Data are from three independent trials, each done in duplicate.

The apparent requirement of intact *prfA* and *hly* for biofilm formation suggested the possibility that colonization of produce might also be impaired in the mutants. Since expression of *hly* and *prfA* is under temperature control and is optimal at 37 °C [10,20], we investigated produce colonization both at 25 °C, a temperature relevant for produce contamination, and at 37 °C. The parental strain and the two mutants showed similar adherence on cantaloupe rind at both 25 and 37 °C. For all three strains, adherent cells represented 35 ± 10% of the population inoculated on the rind. Furthermore, no significant differences in growth on the cantaloupe rind were noted between the parental strain 2858 and either B2G6 or J2E3 at 25 °C (monitored up to 72 h) (Figure 4A). Growth was also not impaired at 37 °C (Figure 4B), a temperature at which, as indicated above, *prfA* and *hly* would be expected to be optimally expressed [10,20].

**Figure 5:**
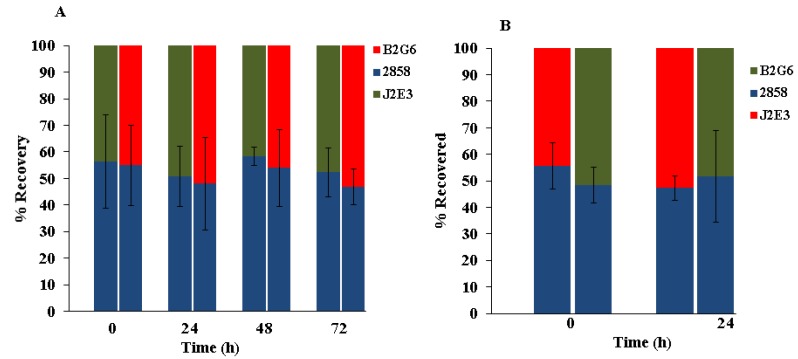
Competitive fitness of *hly* and *prfA* mutants on cantaloupe rind. Cantaloupe fragments were inoculated with 1:1 mixtures of the wildtype parental strain 2858 and each mutant, and inoculated fragments were incubated at (**A**) 25 °C and (**B**) 37 °C. Ratios were determined by sub-culturing colonies from MOX plates in individual wells of 96-well plates and subsequent differentiation on media with and without erythromycin, as described in Materials and Methods. Time 0 corresponds to 30 min after inoculation. Data are from three independent trials, each done in duplicate.

To determine potential impacts of the mutations on the relative fitness of the bacteria on cantaloupe, the rind was inoculated with 1:1 mixtures of the parental strain 2858 and each of the mutants, and incubated at 25 °C (up to 72 h) and 37 °C (24 h). Screening of colonies from the MOX plates on media with and without erythromycin revealed that for both mutants the ratio of parental strain to mutant remained around 50% at all tested time points (Figure 5). Similar data were obtained when randomly-selected colonies from the MOX plates were patched onto blood agar and incubated at 37 °C (data not shown). Such data suggest that relative fitness of the *hly* or the *prfA* mutant on the cantaloupe rind at either 25 or 37 °C did not differ significantly from that of the parental strain.

Surface-specific differences may underlie the observed lack of correlation between biofilm formation and colonization of living plant tissue. Cellulose on the latter, for instance, may facilitate binding of *L. monocytogenes*, e.g., through the *Listeria* cellulose binding protein, Lcp [21]. A similar lack of correlation was found with the *L. monocytogenes* cadmium resistance determinant *cadA4*, which was implicated in biofilm formation but not in colonization of produce [19], and the *L. monocytogenes* penicillin-binding protein *pbp4* [22]. In addition to the composition of the surface, the cantaloupes were all bought at retail and retained their natural microbiota. Interactions with the background microbiota or their metabolites may also have impacted the ability of *L. monocytogenes* to form biofilms.

In conclusion, our analysis of non-hemolytic mutants of strain 2858, a serotype 1/2b strain that contributed to the 2011 listeriosis outbreak via whole cantaloupe, revealed that *prfA* and, to a lesser extent, *hly*, both contribute to the ability of *L. monocytogenes* to form biofilms, and both determinants are involved in aggregation of *L. monocytogenes*. These virulence genes may therefore play important roles in environmental contamination of the produce-packing facilities and food production equipment, thus contributing to food safety risks. However, under the employed conditions neither *prfA* nor *hly* were found to be required for adherence, growth or competitive fitness of *L. monocytogenes* on the cantaloupe rind. Further studies of this and other strains are needed to more fully characterize the involvement of these and other virulence factors in environmental survival and persistence of *L. monocytogenes*, and in contamination of food by this troublesome pathogen.

## 3. Materials and Methods

### 3.1. Bacterial Strains and Growth Conditions

The serotype 1/2b strain 2011L-2858, referred to here as “2858”, was kindly obtained from Cheryl Tarr and was previously investigated for its potential to adhere and grow on cantaloupe [23], as well as the role of a penicillin-binding protein encoded by *pbp4* on copper tolerance and virulence [22]. Strains B2G6 and J2E3 are non-hemolytic mutants of strain 2858 obtained as described below from screening a *mariner*-based mutant library on blood agar (Remel Inc., Lenexa, KS, USA) plates. Unless otherwise indicated, *L. monocytogenes* was grown at 37 °C in brain heart infusion (BHI) broth (Becton, Dickinson & Co., Sparks, MD, USA) or on BHI agar (BHIA, BHI with 1.2% Bacto-agar, Becton, Dickinson & Co.). When needed, erythromycin (MP Biomedicals, Solon, OH, USA) was added at 5 µg/mL in BHI (BHI-Em5) or in BHIA (BHIA-Em5) and kanamycin (Fisher Scientific, Fair Lawn, NJ, USA) was added at 10 µg/mL to BHI (BHI-Km10) and BHIA (BHIA-Km10).

### 3.2. Mutant Library Construction, Screening for Non-Hemolytic Mutants, Determination of Transposon Copy Number and Localization

A *mariner*-based transposon mutant library (approx. 1900 mutants) of strain 2858 was constructed as described using pMC38 [19,24]. Mutants that were confirmed to be erythromycin-resistant (as expected by presence of the transposon) and kanamycin-sensitive (as expected upon loss of the plasmid vector for the transposon) were individually screened on blood agar (Remel Inc., Lenexa, KS, USA) using a sterile 48-pin stainless steel replicator. The plates were incubated at 37 °C for 36 h and examined for hemolysis zones typical of *L. monocytogenes*. The number of transposon insertions in each mutant was determined by Southern blot as described [19,24]. To identify the sites of transposon insertion, arbitrary PCR was performed as described [19,24]. PCR products were sequenced (Genewiz Inc., South Plainfield, NJ, USA), and sequences were analyzed by nucleotide BLAST.

### 3.3. Biofilm Formation and Aggregation Assessments

Procedures previously described [19] were employed to assess biofilm formation in 96-well polystyrene plates (Greiner Bio-One, VWR, Suwanee, GA, USA). Briefly, *L. monocytogenes* strains were grown in wells of 96-well plates at 37 °C for 48 h without agitation. Upon removal of the supernatant, each well was washed three times with deionized water and allowed to air-dry for approx. 45 min. Crystal violet staining, solubilization of the dye in 95% ethanol and biofilm measurements via A_590_ determinations were as described [19]. *L. monocytogenes* was grown in eight wells/strain for each trial, and analyses were done in at least three independent trials. Aggregation assessments were carried out at 37 °C as described previously [13]. Aggregation was calculated by subtracting OD_600_ at 24 h from the OD_600_ at the start of the assay. For microscopy, material from approx. 1 cm below the surface was carefully removed and cells visualized by phase contrast microscopy on a Leica microscope (model LMB 2).

### 3.4. Cantaloupe Rind Adherence and Growth Assessments

Cantaloupes were purchased from local grocery stores in Raleigh, NC and 2 × 2 × 0.5 cm rind fragments were obtained as described [23]. Preparation of inoculum and spot-inoculation of the fragments with 10 evenly separated droplets of 10 µL each (total inoculum, 100 µL) were as described [23]. Adherence and growth assays were also as described [23]. Briefly, inoculated fragments were incubated at the indicated temperature for 1 h, loosely adherent cells were removed by gentle washes, and tightly adhered cells were then removed by vortexing and enumerated by plating dilutions of the rinsate on modified Oxford *Listeria* selective agar (MOX; Oxoid, Hampshire, UK) and incubating at 37 °C for 48 h. To assess growth, petri dishes with inoculated rind fragments were sealed with parafilm and stored for up to 72 h at room temperature (25 °C) or up to 24 h at 37 °C. At specific time points, rinsates of two inoculated cantaloupe fragments per strain were diluted and plated on MOX, followed by incubation for 48 h at 37 °C. Each assessment was done in duplicate, and in at least three independent trials.

### 3.5. Competitive Fitness Assessments

Inoculum for mixed-strain inoculations (1:1 mixture of the wild type parental strain 2858 and each mutant) was prepared by combining cell suspensions corresponding to equal CFUs of each strain. Randomly-chosen colonies from the MOX plates were inoculated individually into 96-well plates containing 200 µL BHI and incubated for approx. 16 h at 37 °C. The 96-well cultures were then stamped using a sterile 48-pin replicator onto BHIA and BHIA-Em5. The ratio of parental strain 2858 (erythromycin-susceptible) and mutant (erythromycin-resistant) was determined following 36 h of incubation at 37 °C. To confirm these findings, randomly-selected colonies from the MOX plates were also streaked on blood agar (Remel, Inc.). The plates were incubated at 37 °C for 36 h and the ratio of hemolytic and non-hemolytic cultures was determined. Each competitive fitness assay was done in at least three independent trials.

### 3.6. Statistical Analysis

For statistical analysis of growth and adherence on produce, including growth in competitive fitness experiments, one-way analysis of variance (ANOVA) with a Tukey’s test was used at *p* < 0.05, with SPSS version 22 (IBM Corporation Software Group, Somers, NY, USA). Biofilm and aggregation data were analyzed using a paired Student’s t-test (*p* < 0.05).

## Supplementary Materials

The following are available online at http://www.mdpi.com/2076-0817/7/1/18/s1, Figure S1: Colony morphology of (A) wild type 2858, (B) *hly* inactivated mutant B2G6, and (C) *prfA* inactivated mutant J2E3; Figure S2: Cell morphology of (A) wild type 2858, (B) *hly* inactivated mutant B2G6, and (C) *prfA* inactivated mutant J2E3; Figure S3 Growth curves of wild type 2858, *hly* inactivated mutant B2G6, and *prfA* inactivated mutant J2E3 at (A) 28 and (B) 37 °C.
