# Supplementary material for: The Listeria monocytogenes Key Virulence Determinants hly and prfA are involved in Biofilm Formation and Aggregation but not Colonization of Fresh Produce
Source: Pathogens. 2018 Feb 1;7(1):18. doi: 10.3390/pathogens7010018 (PMC5874744; doi:10.3390/pathogens7010018)
Supplement: Supplementary file 1 [file pathogens-07-00018-s001.pdf]

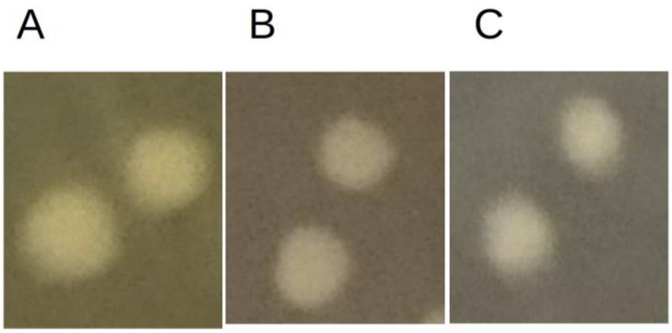

Supplemental Figure 1. Colony morphology of (A) wild type 2858, (B) *hly* inactivated mutant B2G6, and (C) *prfA* inactivated mutant J2E3.

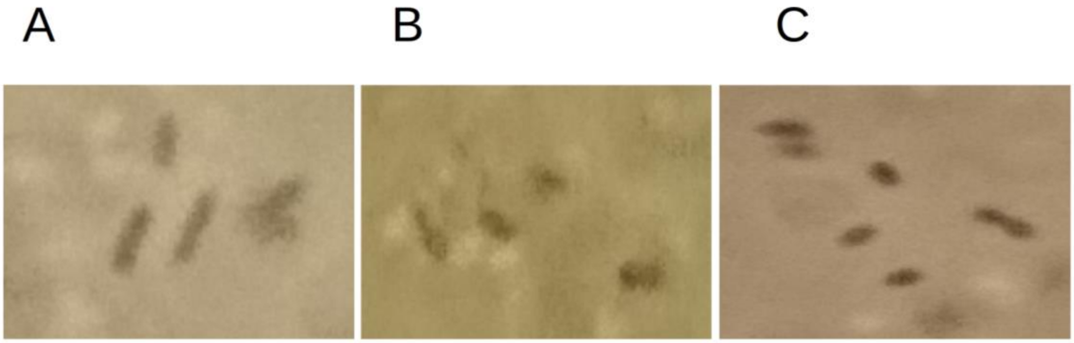

Supplemental Figure 2. Cell morphology of (A) wild type 2858, (B) *hly* inactivated mutant B2G6, and (C) *prfA* inactivated mutant J2E3.

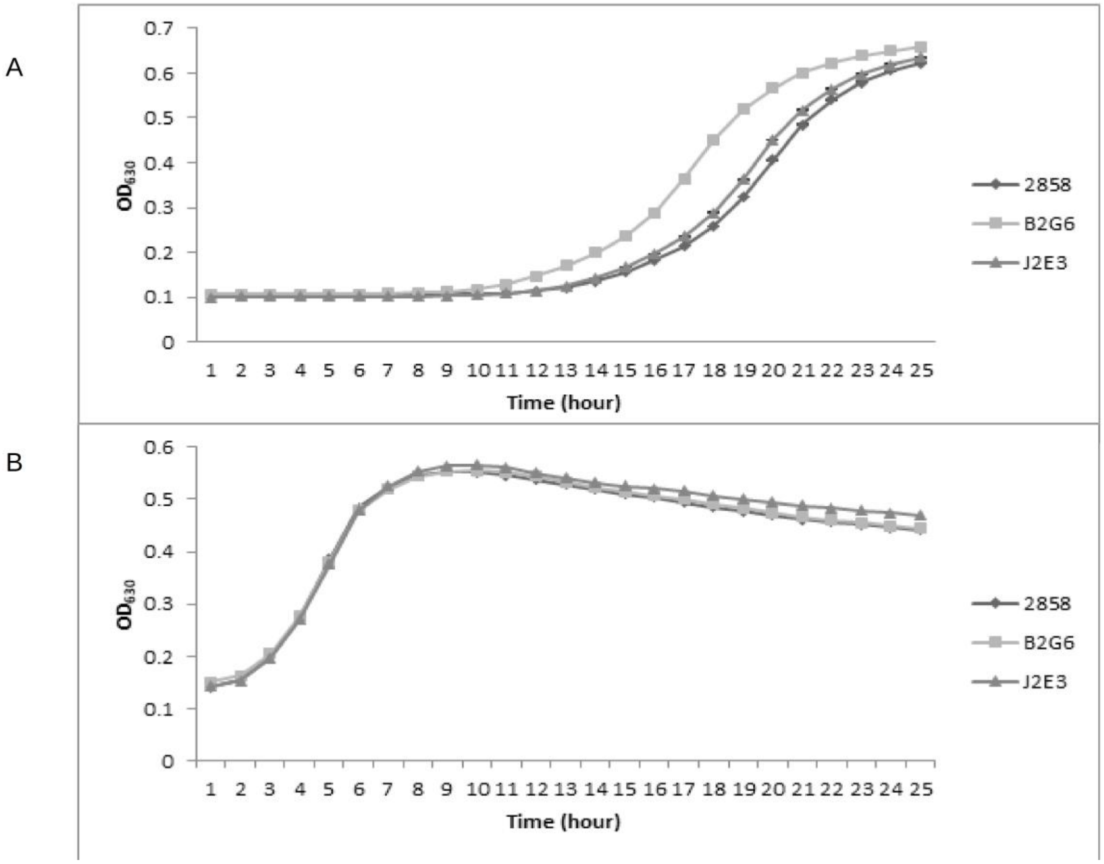

Supplemental Figure 3. Growth curves of wild type 2858, *hly* inactivated mutant B2G6, and p*rfA* inactivated mutant J2E3 at (A) 28 and (B) 37 °C.
